# 3D-analysis of unwanted tooth movements despite bonded orthodontic retainers: a pilot study

**DOI:** 10.1186/s12903-020-01304-2

**Published:** 2020-11-04

**Authors:** Katharina Klaus, Faidra Xirouchaki, Sabine Ruf

**Affiliations:** 1 Department of Orthodontics, Justus-Liebig-University Giessen, Schlangenzahl 14, 35392 Giessen, Germany; 2 Private Practice, Ethnomartiron 70A, 71409 Heraklion (Iraclio), Crete Greece

**Keywords:** Lingual retainer, Fixed retainer, Unexpected tooth movement

## Abstract

**Background:**

Recently, reports of unwanted tooth movements despite intact orthodontic bonded retainers have increased. These movements are not subject to relapse but are classified as a new developed malocclusion. The aims of the present pilot study were to analyze the prevalence of unwanted tooth movements despite intact bonded cuspid-to-cuspid retainers and to identify possible predisposing factors.

**Materials and methods:**

Plaster casts of all patients finishing orthodontic treatment during three consecutive years were assessed before treatment (T0), after multibracket appliance debonding (T1) and after two years of retention (T2). After multibracket appliance treatment, all patients received a cuspid-to-cuspid flexible spiral wire retainer bonded to each tooth of the retained segment in the upper and lower jaw. The study group (SG) consisted of 44 patients (16 male, 28 female) with tooth movements (T1–T2) of the retained segment despite intact bonded cuspid-to-cuspid retainer and the control group (CG) of 43 patients (19 male, 24 female) without unwanted tooth movements. The casts of the SG were digitized, superimposed and measured. Using the Chi-square test, Fisher´s exact test and Mann–Whitney-U-test (p < 0.05), mandibular plane angle, incisor proclination, oral dysfunctions or habits (T0) and intercanine distance, overjet and interincisal relationship (T0, T1, T2) were compared between SG and CG.

**Results:**

The prevalence of patients with unwanted tooth movements in one or both jaws was 27.0%. Maxillary retainers were affected more often (20.9%) than mandibular retainers (14.1%). The median amount of tooth movements was 0 to 0.66 mm with large interindividual variations. Oral dysfunctions or habits at T0, such as a lack of interincisal contact at all time points, were associated with unwanted tooth movements.

**Conclusion:**

Unwanted tooth movements occurred more often with maxillary than mandibular retainers. Patients with oral dysfunctions/habits and without interincisal contact had a higher prevalence of unwanted tooth movements.

## Background

Maintaining orthodontic treatment results long term is a challenging task in orthodontics. To prevent relapse tendencies and aging processes, lifelong retention seems to be necessary [1]

Since their introduction in the 1970s, bonded lingual retainers have grown in popularity and survey studies in different countries have shown their preference for long-term retention after orthodontic treatment in the mandible [2, 3] or in both arches [4–7]. Many studies revealed the high reliability of fixed retainers bonded to the lingual surfaces of all six anterior teeth in maintaining incisor alignment [8–14]. However, unexpected tooth movements despite intact bonded retainers may occur [11, 15–24]. Some articles on this subject are however case reports or small case series; thus, information regarding the incidence of such side effects is rare and ranges between 1.1 and 43.3% of the patients [11, 16, 19]. In some questionnaire-based survey studies, unwanted side effects were reported by 56.7 to 100% of respondent orthodontists, however only a small number of patients were involved [4, 5, 7].

Different patterns of unwanted tooth movements have been described: torque differences between two adjacent incisors, opposite torque of canines, and increased buccal or lingual inclination of single canines [11, 16, 19, 22, 23]. These movements are classified as newly developed posttreatment malocclusion rather than relapse, because they show no similarities to the pretreatment malocclusion [11, 16, 22]. Such movements are attributed to an activation/distortion of the retainer wire, with the underlying mechanisms being yet unknown. Until now, only two studies have investigated this subject systematically for the lower jaw [19, 22]. Kucera and Marek [22] screened a total of 3500 patients at their annual retention recall and reported that 1.1% of them were affected by unwanted tooth movements. Compared to patients without such complications, the affected subjects were younger at debonding and had higher pretreatment mandibular plane angles (ML/NSL) and increased pretreatment incisor proclination relative to the A-Pogonion line (Ii-APo). Wolf et al. [19] investigated 30 consecutively-treated patients and detected moderate or severe tooth movements in 43.3% of them. Using digital superimpositions of the casts, they found larger unwanted tooth movements in cases with larger intercanine expansion and larger overjet reduction during treatment.

Due to the limited number of systematic papers, the large span of reported prevalence and tooth movement patterns as well as the fact that unexpected tooth movements in the maxilla have not been investigated previously, the present retrospective study aimed to:

- detect the prevalence of unexpected tooth movements despite bonded retainers in the upper and lower jaw in a university orthodontic department,
- visualize and measure the amount of unexpected tooth movements, and
- identify possible pretreatment and/or treatment-related predisposing factors.

## Materials and methods

The study was approved by the ethics committee of the medical faculty of the Justus-Liebig-University Giessen, Germany (number 71/18).

All patients who completed active orthodontic treatment and a supervised retention period of approximately two years at the Department of Orthodontics of the Justus-Liebig-University Giessen, Germany, over three consecutive years (2010—2012) were assessed. This time span was chosen because in 2005 the head of the department decided to change the retainer type for patients scheduled for fixed retention from cuspid retainers with two bonding sites at the cuspids to retainers bonded on all six anterior teeth. Therefore, five to seven years (2010–2012) after this decision, the majority of completed treatments should have had cuspid-to-cuspid retainers with six bonding sites. The further inclusion criteria were:

1. multibracket appliance treatment in both jaws,
2. fixed cuspid-to-cuspid retainers, bonded on all six teeth (canines, lateral and central incisors) in the upper and lower jaw,
3. perfectly intact plaster casts from three different time points: pretreatment (T0), after debonding of the multibracket appliance (T1) and after the supervised retention period of approximately two years (T2),
4. complete cuspid-to-cuspid retainers in situ at T2.
5. no active orthodontic intervention/retreatment for any reason during the retention period.

Patients were excluded if they had additional removable retainers, bonded retainers including other teeth than the canines and the lateral and central incisors, e.g. due to space closure in cases of missing incisors. Patients who received adhesive or prosthetic restorations of the canines and incisors between T1 and T2 were also excluded.

All retainers were fabricated by a dental technician of 0.018 inch 6-strand coaxial wire (Dentaflex®, Dentaurum, Ispringen, Germany) and bonded by the orthodontists and residents of the department using the direct bonding method according to Zachrisson and Büyükyilmaz [25] using Transbond XT™ or Transbond LR™ (3 M Unitek, Monrovia, California, USA). During the supervised retention period, the control appointments were scheduled three months and six months after debonding of the multibracket appliance and further biannually until active treatment was completed two years after debonding. In case of complications (detachment of bonding sites/wire breakages), patients were advised to arrange an appointment immediately. Detached bonding sites were rebonded. In 9 cases, wire breakages were repaired by chairside adaptation and bonding of a second bridging wire (same material as the initial retainer) between the two neighboring teeth affected by the wire breakage.

One investigator (FX) studied the plaster casts of all patients who met the inclusion criteria. All T1 and T2 casts were visually inspected regarding unwanted tooth movement patterns previously described in the literature [11, 16, 19, 22, 23] or other differences in alignment or tooth position by comparing the incisal edges and marginal ridges of the retained segment including the neighboring premolars. All plaster casts with even small uncertainties and those with suspected tooth movements between T1 and T2 were additionally judged by two experienced orthodontists (KK, SR). In uncertain cases, the incisal edges and marginal ridges were colored using a soft pencil to reach consensus. All patients with agreement regarding unwanted tooth movements were assigned to the study group, while the patients without tooth movements and suitable casts of the upper and lower jaw were assigned to the control group. The plaster casts (T1, T2) of the study group were digitized with a desktop scanner (OrthoX®, Dentaurum, Ispringen, Germany) and saved as Standard Tesselation Language (STL) files. The scans of T1 and T2 were superimposed using the software GOM Inspect 2017 (GOM GmbH, Braunschweig, Germany). In the maxilla, a mushroom-shaped area comprising the stable structures of the hard palate [26] was selected and used for best-fit-superimposition. Due to the lack of stable structures in the mandible, the crowns of all present premolars and the first molars were used for best-fit-superimposition. In non-extraction cases, the first and second premolars and the first molars were used, while in extraction or agenesis cases, the remaining premolars and first molars were used. The second molars could not be used for superimposition because of their ongoing eruption between T1 and T2 in some cases.

**Fig. 1:**
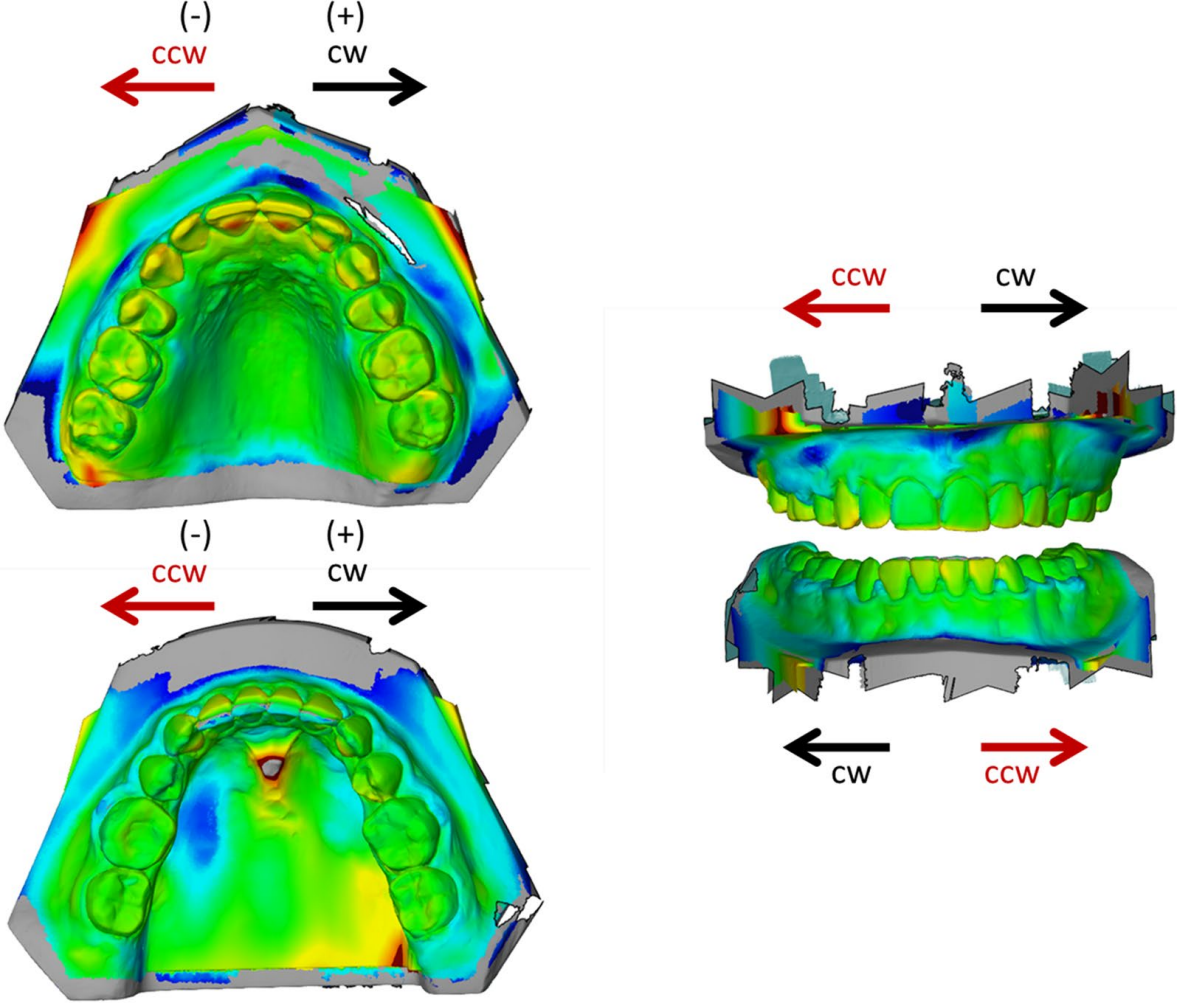
Orientation of digital casts for assessment of tooth movements in transverse direction. *cw* clockwise, *ccw* counterclockwise

In a second step, a surface comparison was performed and canines and lateral and central incisors, which had moved between T1 and T2, were identified. The amount of movement was measured in all three planes of space: sagittal (protrusive/retrusive), vertical (extrusive/intrusive) and transverse (clockwise/counterclockwise) using the software GOM Inspect. For a clockwise (cw)/counterclockwise (ccw) movement assessment, the upper and lower arches were oriented with the incisors facing the top and the molars facing the bottom of the screen (cw = ( +), ccw = (−), Fig. 1). All digital superimpositions and measurements were performed by one single investigator (KK).

The values for pretreatment mandibular plane angle (ML/NSL) and incisor proclination (upper incisor proclination angle (IsL/NA), lower incisor proclination angle (IiL/NB), lower incisor proclination relative to the A-Pogonion line (Ii-Apo)) were recorded from the pretreatment lateral cephalograms. The presence of oral dysfunctions or habits (tongue thrusting, tongue thrust swallowing, sucking habits and/or a resting position of the lower lip behind the upper incisors) were retrieved from the patient records. Furthermore, the complication rates (debondings, wire breakages) during the retention period were recorded.

From the plaster casts at T0, T1 and T2, the overjet and the intercanine distance in both jaws were measured with a manual caliper (Zürcher Modell, Karl Hammacher GmbH, Solingen, Germany), because the initial T0 casts had not been digitized. Furthermore, the interincisal relationship was assessed and categorized (incisor overlap with interincisal contact/incisor overlap without interincisal contact/open bite). Measurements were performed to the nearest 0.5 mm.

### Statistical analysis

Statistical analysis was performed by a professional medical statistician using SPSS Statistics 25 (SPSS Inc. an IBM Company, Chicago, IL, USA). Due to the nature of a pilot study, no sample size calculation was performed a priori.

The data did not show a normal distribution (Kolmogorov–Smirnov-test, Shapiro–Wilk-test). In addition to descriptive statistics, the Chi-square test and the Fisher´s exact test were used for the comparison between tooth movement and dichotomous variables. The comparison between tooth movement and pretreatment or treatment-related quantitative variables was performed by the Mann–Whitney-U-test. The significance level was set at 0.05.

To assess the method reliability regarding digital superimpositions and measurements, 15% of the scans were superimposed and measured for a second time by the same investigator at least 4 weeks after the first evaluation. The consistency between the measurements was determined using intraclass correlation coefficients (ICC). For both digital superimpositions (ICC = 0.925) and measurements (sagittal: ICC = 0.996, vertical: ICC = 0.994, transverse: ICC = 0.961), excellent reliability was found.

## Results

**Fig. 2:**
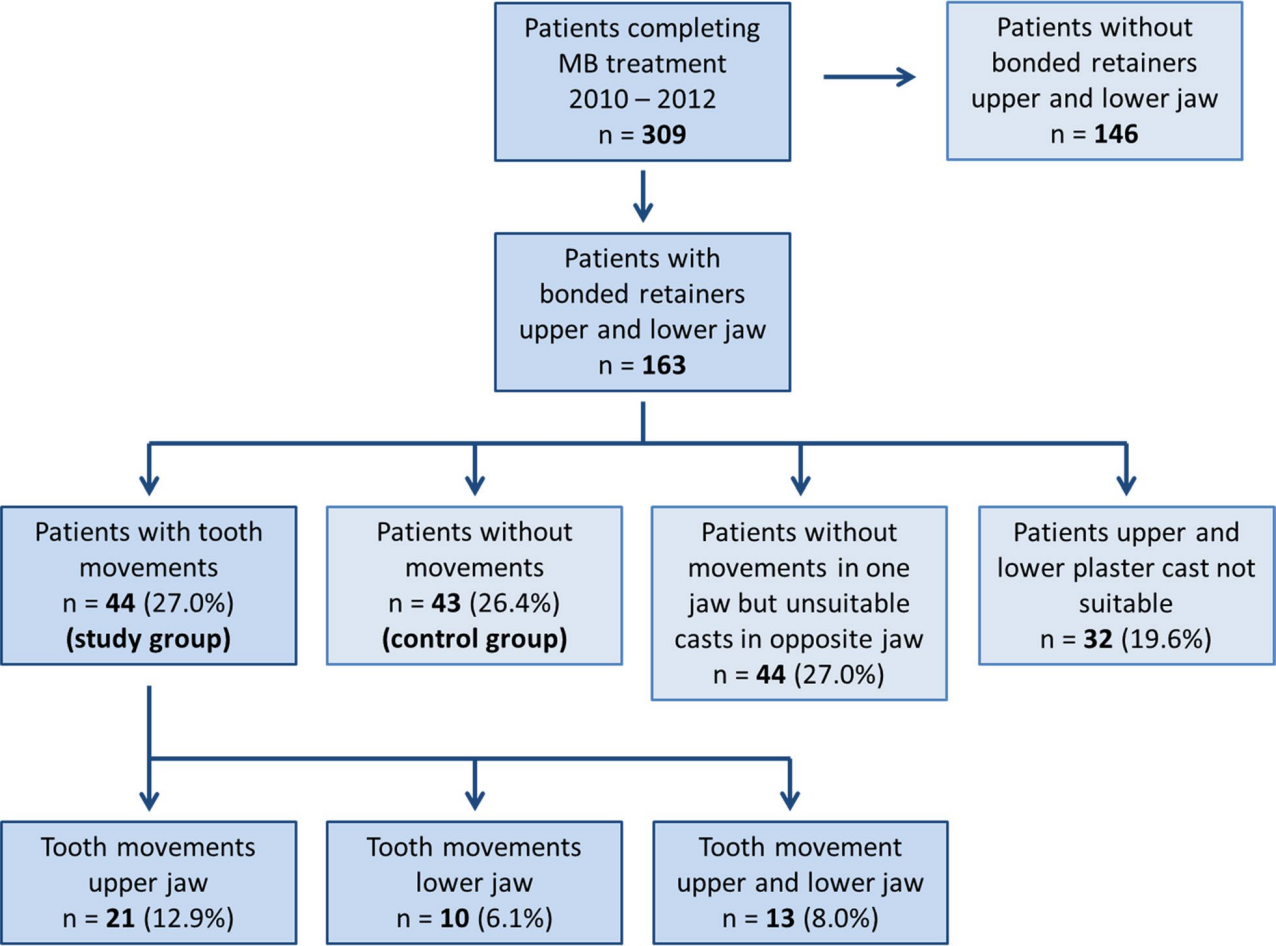
Study population flowchart

The flowchart of the study population is given in Fig. 2. The descriptive characteristics of the study group and the control group are displayed in Table 1.

**Table 1:** Descriptive characteristics of patients affected by unwanted tooth movements (study group) and nonaffected patients (control group)

	Study group (n = 44)		Control group (n = 43)		p-value
	n	%	n	%	
Female	28	63.6	24	55.8	0.457
Male	16	36.4	19	44.2	0.457
Angle class I	22	50.0	21	48.8	0.914
Angle class II	20	45.5	17	39.5	0.577
Angle class III	2	4.5	5	11.7	0.225
Extractions	15	34.1	13	30.2	0.700

	Mean (yrs)	SD	Mean (yrs)	SD	
Age at T0	12.68	2.11	14.36	5.60	0.136
Age at T1	15.44	2.20	16.97	5.61	0.327
Age at T2	17.32	2.74	19.13	5.58	0.231
Treatment duration	2.73	0.70	2.61	0.82	0.236
Retention phase	1.89	1.57	2.15	0.41	0.706

*n* number, *yrs *years, *SD* standard deviation

During the three consecutive years (2010–2012), a total of 309 patients completed multibracket appliance treatment and a supervised retention period of approximately two years. Bonded retainers in the upper and lower arch were inserted in 163 patients. In 27% (n = 44 patients; 28 female, 16 male) an unexpected tooth movement in one or both arches occurred, while in 26.4% of patients, no unintentional tooth movement was seen. In 27% of the patients, there was no unwanted tooth movement in one jaw, while the plaster cast of the opposite jaw was not suitable for evaluation. In 19.6% of the patients, both upper and lower plaster casts were not suitable for evaluation because of missing casts, obviously distorted areas, plaster fractures or removal of bonded retainers ahead of schedule and thus no certainty with respect to unwanted tooth movements.

Within the group with unwanted tooth movements, 13 patients had movements in the upper as well as in the lower jaw. Maxillary retainers were affected more often (n = 34, 20.9%) than mandibular retainers (n = 23, 14.1%).

**Fig. 3:**
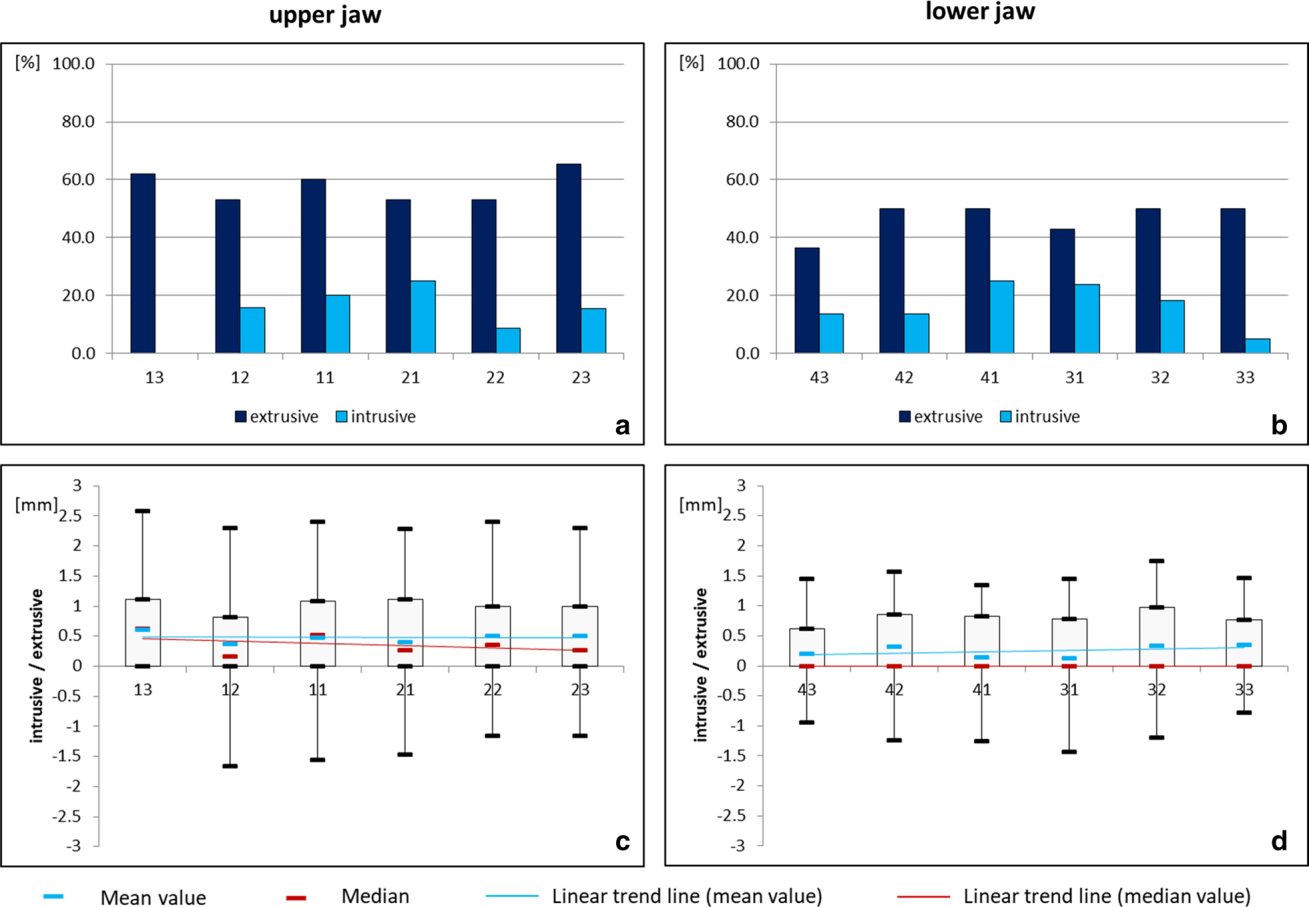
Vertical movement of upper and lower teeth under fixed retention (study group). Relative frequency (**a**, **b**) and amount (**c**, **d**) of extrusive (+) and intrusive (−) movements in the upper and lower jaw

In the vertical plane, extrusive tooth movements prevailed (Fig. 3). Upper teeth were more frequently affected than lower ones. The interquartile range showed an even distribution except for tooth 13. The median for all upper and lower teeth ranged between 0 and 0.62 mm with small and clinically irrelevant differences between the teeth. Although, large interindividual variations were observed, ranging from 2.58 mm extrusion for tooth 13 to 1.66 mm intrusion for tooth 12 in the upper jaw and from 1.75 mm extrusion for tooth 32 to 1.43 mm intrusion for tooth 31 in the lower jaw. However, it should be noted that especially for extrusive movements, a distinction between post-orthodontic settling and unwanted tooth movements cannot be made.

**Fig. 4:**
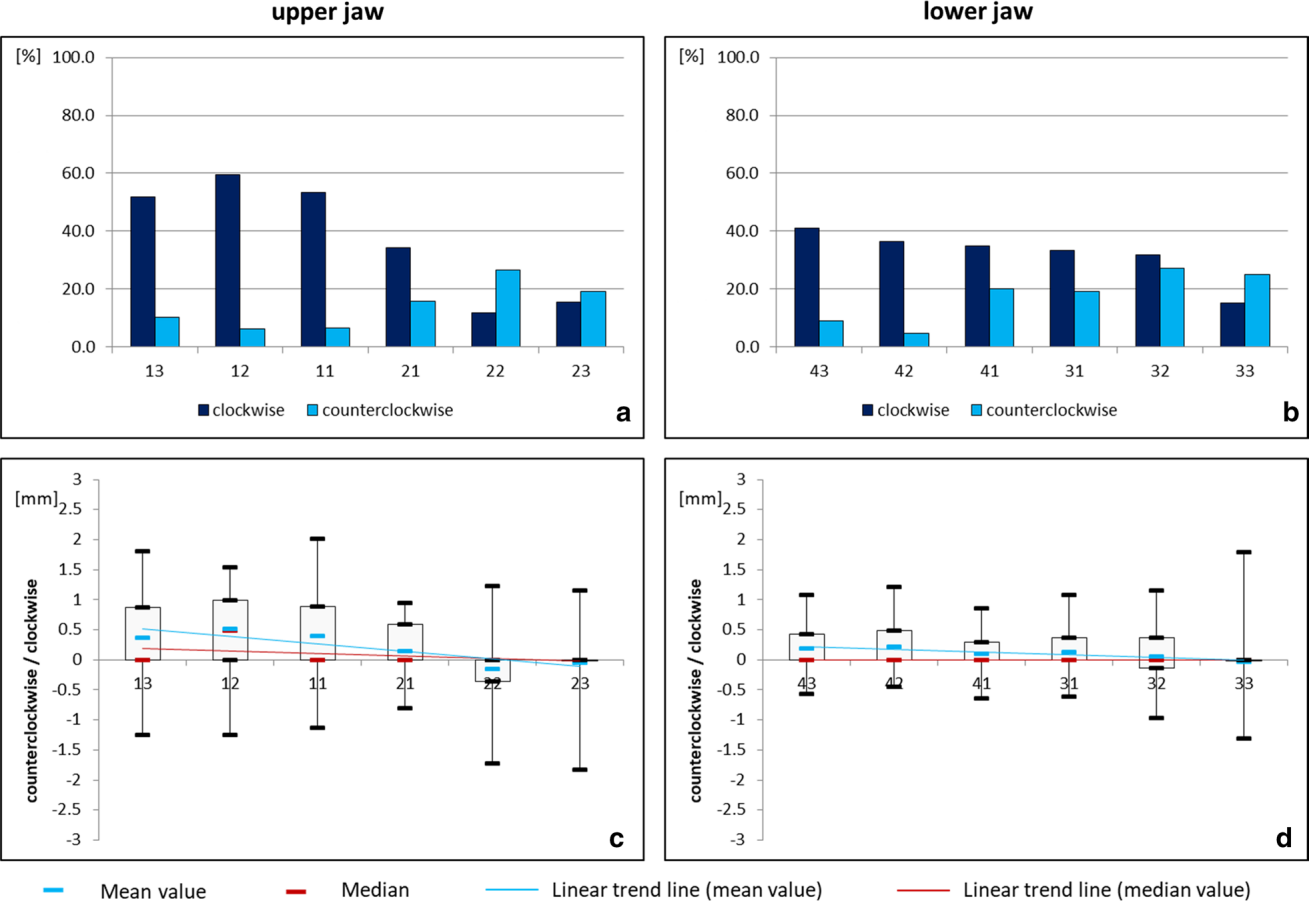
Transverse movement of upper and lower teeth under fixed retention (study group). Relative frequency (**a**, **b**) and amount (**c**, **d**) of clockwise (+) and counterclockwise (−) movements in the upper and lower jaw

In the transverse dimension, a clockwise movement was most commonly seen (Fig. 4). The median ranged between 0 and 0.49 mm. Again, large interindividual variations occurred, reaching from 2.02 mm clockwise movement of tooth 11 to 1.84 mm counterclockwise movement of tooth 23 in the maxilla and from 1.79 mm clockwise to 1.31 mm counterclockwise movement of tooth 33 in the mandible. The teeth in the patient´s first and fourth quadrant seemed to be most affected as indicated by the trend line. This trend was again more pronounced in the maxilla than the mandible.

**Fig. 5:**
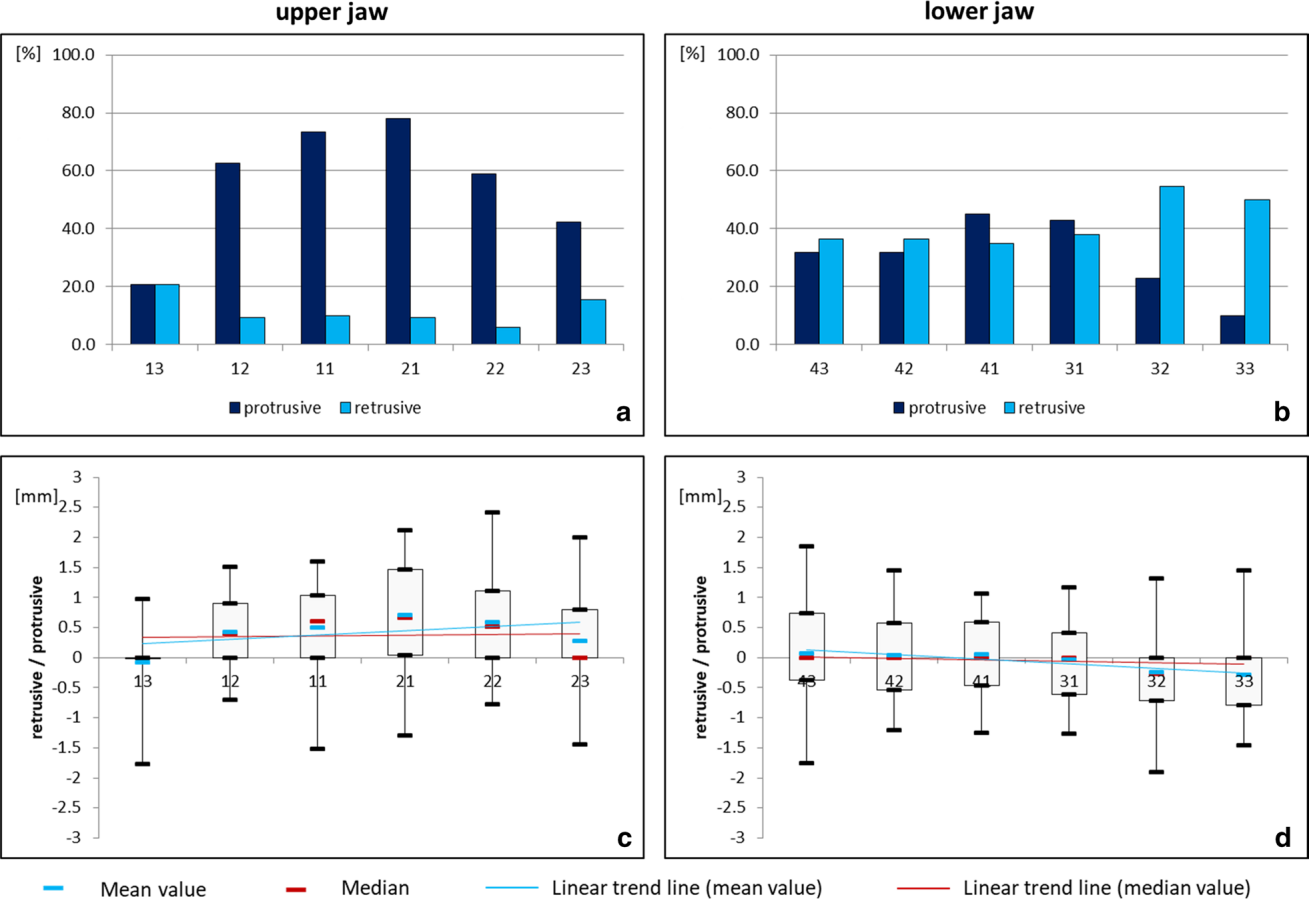
Sagittal movement of upper and lower teeth under fixed retention (study group). Relative frequency (**a**, **b**) and amount (**c**, **d**) of protrusive (+) and retrusive (−) movements in the upper and lower jaw

In the sagittal plane, mainly protrusive movements occurred in the upper jaw, while both protrusive and retrusive movements were found in the lower jaw (Fig. 5). The median ranged between 0 and 0.66 mm. The interindividual variation ranged between 2.42 mm protrusive movement of tooth 22 to 1.77 mm retrusive movement of tooth 13 in the upper jaw and from 1.85 mm protrusive movement of tooth 43 to 1.9 mm retrusive movement of tooth 32 in the lower jaw. In the maxilla, the incisors showed the most pronounced protrusion, whereas in the mandible a tendency towards an opposite inclination of the canines prevailed.

The analysis of pretreatment- and treatment-related predisposing factors indicated that patients with persistent oral dysfunctions or habits prior to orthodontic treatment were more often affected by unwanted tooth movements (p = 0.049). The interincisal relationship presented statistically significant differences as well. The patients with unwanted tooth movements were more likely to have an incisal overlap without interincisal contact at all time points (p < 0.01; Table 2), whereas the control group was more likely to have an interincisal contact at all time points (p < 0.01; Table 2).

**Table 2:** Pretreatment oral dysfunctions/habits and incisal relationship of patients affected by unwanted tooth movements (study group) and nonaffected patients (control group)

	Study group (n = 44)		Control group (n = 43)		p-value
	n	%	n	%	
*Oral dysfunctions/habits at T0*	19	43.2	10	23.3	0.049
*Interincisal relationship at T0*					
Incisal overlap with interincisal contact	17	38.6	35	81.4	< 0.001
Incisal overlap without contact	26	59.1	6	14.0	< 0.001
Open bite	1	2.3	2	4.7	0.543
*Interincisal relationship at T1*					
Incisal overlap with interincisal contact	15	34.1	27	62.8	0.007
Incisal overlap without contact	29	65.9	16	37.2	0.007
Open bite	0	0.0	0	0.0	1
*Interincisal relationship at T2*					
Incisal overlap with interincisal contact	17	38.6	31	72.1	0.002
Incisal overlap without contact	26	59.1	11	25.6	0.002
Open bite	1	2.3	1	2.3	0.987

*n* number

Neither the pretreatment mandibular plane angle, the amount of incisor proclination, the degree of expansion of the intercanine distance nor the overjet reduction during treatment showed a statistically significant association with unintentional tooth movements (Table 3).

**Table 3:** Pretreatment mandibular plane angle/incisor proclination and treatment-related alterations of intercanine distance and overjet of patients affected by unwanted tooth movements (study group) and nonaffected patients (control group)

Means of pretreatment values/treatment related alterations	Study group (n = 44)	Control group (n = 43)	p-value
Mandibular plane angle (ML/NSL) [°]	33.8	32.0	0.157
*Incisor proclination*			
Upper incisor proclination angle (IsL/NA) [°]	22.8	20.7	0.650
Lower incisor proclination angle (IiL/NB) [°]	24.8	24.1	0.675
Lower incisor proclination (Ii-Apo) [mm]	0.99	0.58	0.832
*Intercanine distance T1–T0 [mm]*			
Upper jaw	1.55	0.66	0.352
Lower jaw	0.00	0.34	0.432
*Overjet T1–T0 [mm]*	−2.65	−2.47	0.571

*n* = number

The same was true for the detachment of bonding sites and wire breakages (Table 4), which were only seen in the upper jaw, during the supervised retention period.

**Table 4:** Number of bonding site detachments per tooth and number of wire breakages per interdental area (location) of patients affected by unwanted tooth movements (study group) and nonaffected patients (control group). Wire breakages were seen only in the upper jaw

	Study group (n = 44)		Control group (n = 43)		p-value
	n	%	n	%	
*Bonding site detachment (tooth)*					
13	7	15.9	7	16.3	0.963
12	12	27.3	9	20.9	0.489
11	10	22.7	6	14.0	0.291
21	7	15.9	5	11.6	0.563
22	11	25.0	9	20.9	0.652
23	8	18.2	9	20.9	0.747
33	8	18.2	5	11.6	0.391
32	10	22.7	5	11.6	0.171
31	6	13.6	8	18.6	0.528
41	10	22.7	4	9.3	0.088
42	11	25.0	5	11.6	0.107
43	11	25.0	5	11.6	0.107
*Wire breakage* *(location)*					
13/12	2	4.5	0	0.0	0.157
12/11	0	0.0	0	0.0	1
11/21	3	6.8	0	0.0	0.081
21/22	1	2.3	0	0.0	0.320
22/23	2	4.5	1	2.3	0.157

*n* number

## Discussion

### Prevalence of unexpected tooth movements

In the present study sample, 27% of the patients showed unwanted tooth movements despite intact bonded retainers in the upper and/or lower jaw. Unwanted tooth movements exclusively in the mandible were seen in 14.1% of the subjects. In the literature, this complication has only been described for the lower jaw with prevalence rates ranging between 1.1 to 43.3% [11, 16, 19]. Thus, our patient sample is in the lower span of the reported prevalence.

The study describing the highest prevalence of 43.3% [19] categorized the samples into "stable," "moderate" and "severe" cases based on the amount of rotational position changes. They found that 30% of the patients were moderately affected and 13.3% were severely affected, justifying retreatment for the severe group. In comparison, all patients in the present study completed the entire retention period without unwanted tooth movements being classified as severe enough to justify retreatment. The severely affected cases corresponding to the 13.3% of the sample by Wolf et al. [19] were initially excluded in the present study because of retreatment during the retention phase. Taking this into account, the prevalence rate in the present study underestimates the phenomenon.

In the present sample, the maxillary retainers were affected more often (20.9%) than the lower ones. To date, there are no other studies in the literature analyzing upper retainers. This may be due to the fact that bonded retainers are not used that commonly in the upper jaw, as indicated by questionnaire-based survey studies [2, 3, 7]. Furthermore, survey studies and survival studies regarding upper bonded retainers show a large variety in different retainer extensions for the upper jaw: 1–1 retainers (only the central incisors), 2–2 retainers (central and lateral incisors) and 3–3 retainers (canines, central and lateral incisors) [4, 6, 27–30]. If maxillary retainers are extended to the canines as in the present study, a higher incidence of retainer losses and wire breakages is reported [25, 27, 30], especially in deep bite cases. Although all upper retainers in the present study were initially placed out of occlusion, a gradual bite deepening during the retention period is often observed clinically, whether caused by settling of the occlusion or an initial relapse of deep bite correction [31–33]. Potentially, such a contact of the lower canines on the retainer wire between the upper laterals and the canines could have resulted in distorting the retainer wire, explaining some of the observed unwanted tooth movements in the upper jaw.

### Direction and amount of unexpected tooth movements

In the vertical plane, predominantly extrusive movements occurred in both arches, though the upper teeth were more frequently and more severely affected than the lower ones. In the current literature, only Wolf et al. [19] measured the amount of unexpected movements in the lower arch, but due to methodological differences, the comparability to the present results is limited. Wolf et al. [19] present a mean apicocoronal movement for the severely affected lower canines of 0.52 ± 0.35 mm, which is larger than the median values of the present samples (Fig. 3). However, the present samples did not comprise severely affected cases requiring retreatment as mentioned above and could thus underestimate the amount of tooth movement. Especially for the vertical dimension, the extrusive movements could also have been caused by post-orthodontic settling alone. Theoretically, a distinction of post-orthodontic settling and unwanted extrusive tooth movements for the upper jaw could be investigated by measuring extrusive movements of the premolars and molars and subtracting the amount of extrusion of the lateral teeth from the amount of extrusion of the retained teeth, because the jaws were superimposed using the stable structures of the hard palate. Due to the lack of stable structures in the mandible and the necessity for superimposition on dental structures, a distinction between post-orthodontic settling and unwanted tooth movements in the lower jaw is not possible.

In the transverse dimension, clockwise movements prevailed. Again, the upper jaw was more frequently and more severely affected than the lower jaw. Furthermore, the teeth of the patient´s first and fourth quadrant seemed to be more affected than the teeth of the second and third quadrant. As previously suspected by Kucera and Marek [22], asymmetric unwanted tooth movements could possibly be due to the winding/unwinding direction of multistranded retainer wires. However, this hypothesis remains scientifically untested.

In the sagittal plane, the upper teeth showed mainly protrusive movements, which were more frequent and more severe in the incisors compared to the canines. This could be due to a transverse relapse, resulting in an extension of the dental arch. However, the analysis of treatment-related factors revealed no association between alterations of the intercanine distance and unintentional tooth movements. In the lower jaw, the movement pattern of the present sample shows similarities to the twist effect previously described in the literature [11, 19, 22, 23].

### Pretreatment or treatment-related factors

In contrast to Kucera and Marek [22], neither the mandibular plane angle nor the incisor proclination before treatment was correlated to unintentional tooth movements. Wolf et al. [19] found patients with larger amounts of intercanine expansion and greater overjet reduction during orthodontic treatment to be more affected by severe posttreatment tooth movements in the lower jaw. This could not be confirmed by the results of the present study.

In the present sample, affected patients presented oral dysfunctions or habits prior to orthodontic treatment more often. Additionally, for all time points, a significant association with the interincisal relationship was observed: affected patients presented an incisor overlap without interincisal contact more frequent, while unaffected patients presented an interincisal contact more often. This factor has not been investigated previously. Theoretically, the inability to establish an interincisal contact even during MB treatment could be due to an abnormal resting position of the tongue and tongue thrust swallowing, which could result in orovestibular-directed forces created by the tongue, and in turn, unintentional tooth movement of the retained segment.

Another possible explanation for unwanted tooth movements despite bonded retainers could be an iatrogenic activation of the retainer during bonding, as proposed by some authors [17–19, 22, 23]. However, in that case, tooth position changes should occur within a few weeks after bonding. In contrast, in the present sample as well as in other study samples [11, 16, 18, 22], later onsets of tooth position changes are reported. These changes could be the result of wire fatigue or deformation caused by masticatory forces or hard food particles [16, 18, 22].

Some in-vitro studies investigated the mechanical properties of retainer wires: Sifakakis et al. [34] simulated intrusive-extrusive and labio-lingual bite forces. They found even small wire displacements of 0.2 mm could exert forces of approximately 1 N on the teeth, which would be sufficient to cause unwanted tooth movements. Another in vitro study [35] investigated retainer wires loaded with intrusive forces, which resulted in residual forces and moments in all retainer types, thus demonstrating that retainer wires are not passive after loading and may induce tooth movements.

### Limitations of the study

In the present study, 46.6% of the patients had to be excluded because of unsuitable plaster casts. Due to the methodology, perfectly intact plaster casts for further scanning, superimposition and measurement were necessary to prevent method errors. Furthermore, patients with bonded retainers including teeth other than the canines, lateral and central incisors as well as patients with adhesive or prosthetic restorations were excluded. Especially regarding the prevalence of unexpected tooth movements found in the present study, the high drop-out rate has to be taken into account.

Additionally, the retainers were not bonded by one single operator but by all orthodontists and residents of the department following the same protocol. While two investigations found operator experience to be a significant factor regarding detachments of bonding sites [30, 36], Lie Sam Foek et al. [37] could not detect any association. Due to the fact that operator experience has not been studied in cases of unwanted tooth movements despite intact bonded retainers, the impact can only be hypothesized. Nevertheless, further prospective studies should aim at the highest possible standardization for retainer bonding.

Complications during the supervised retention period (detachments of bonding sites, wire breakages) showed no statistically significant association to unexpected tooth movements, however they occurred only in some patients, limiting the statistical power. Nevertheless, their impact on the registered unwanted tooth movements cannot be totally ruled out. In some cases, patients were unaware of detached bonding sites until these were observed by the practitioner at the control appointment. During such a period, tooth movements may occur. To overcome this limitation, affected patients should have been excluded from the study. Unfortunately, this was not possible due to the fact that the exact time points of detachment were not documented.

Another possible limitation could be seen in the method of digital superimposition. In the maxilla, the hard palate area around the third rugae has been used as a stable structure for digital superimposition in several studies [26, 38–42]. Despite the fact that this area is not absolutely stable due to growth and remodeling [43, 44] as well as orthodontic treatment using rapid maxillary expansion or maxillary protraction headgear [45], stability seems acceptable for intervals comprising up to two years [42], especially during a retention period as in the present sample. Furthermore, a recently published systematic review confirms that this area is the most accurate one for maxillary superimposition to date [46]. For mandibular superimposition, the most actual approaches use CBCT data [47–50], which is however ethically unacceptable for orthodontic retention surveillance. Only one study [51] investigated the superimposition of different anatomic mandibular areas depicted on digital casts and found acceptable accuracy in patients with bilateral mandibular tori only. Due to the fact that the prevalence of mandibular tori is only about 25% and varies with ethnicity [52, 53], to date there is no reliable methodology for superimposition using stable structures in the mandible [46]. Therefore, as in the study by Wolf et al. [19], a dental superimposition was used. The limitations arising from a dental superimposition, such as changes in tooth position due to post-orthodontic settling and dentofacial growth as well as changes in the tooth morphology due to abrasion, attrition and/or erosion, must be acknowledged.

Digital dentistry, and especially the possibilities of model superimposition and measurement, is a fast-growing field and current subject of research. Due to the present limitations of superimposition discussed above and the fact that the topic of unwanted tooth movements despite intact bonded retainers is not yet well investigated, to date there are no scientifically based thresholds or cut-off values identifying unwanted tooth movements. Therefore, the present pilot study approach used a visual inspection of plaster casts initially, as visually recognized tooth movements could also be recognized by patients, general dentists or orthodontists during routine appointments and therefore may be considered clinically relevant.

In the present study, all measurements in the sagittal, vertical and transverse dimension were performed manually using the software GOM Inspect. Despite the excellent ICCs, the manual identification of landmarks bears a risk for errors and operator bias, especially if only one investigator performs all measurements, as in the present study. Other studies used software-aided measurement algorithms, which can express rotational and translational tooth movements along the x-, y- and z-axes of a coordinate system [19, 42]. To minimize individual errors and enhance comparability to other studies, the use of an alternative software that allows for superimposition and automated measurements could be beneficial.

In summary, the phenomenon of unintentional tooth movements despite bonded retainers still lacks a solid research background. To our knowledge, the present study is only the third systematic retrospective study on the topic and the first one including the upper jaw. Prevalence, extent and etiology remain to be elucidated by further research projects.

## Conclusion

From the results of the present study, the following conclusions were drawn:

- 27% of patients showed unexpected tooth movements despite bonded retainers in the upper and/or lower jaw.
- Maxillary retainers were affected more often than mandibular retainers.
- The median amount of tooth movements in the three planes of space was only 0 to 0.66 mm, but large interindividual variations up to 2.58 mm were seen.
- Patients with oral dysfunctions or habits prior to orthodontic treatment and with incisal overlap without interincisal contact had a higher prevalence of unwanted tooth movements.

In clinical practice, bonded retainers should be supervised carefully as long as they are left in place. Scientifically, the phenomenon of unwanted tooth movements despite fixed bonded retainers should be subject to further studies.

## Data Availability

The datasets supporting the conclusions of this article are available from the corresponding author on reasonable request.

## Abbreviations

SG: Study group
CG: Control group
T0: Timepoint pretreatment
T1: Timepoint after debonding of multibracket appliance
T2: Timepoint after two years of retention
ML/NSL: Mandibular plane angle
Ii-Apo: Lower incisor proclination relative to the Point A-Pogonion line
STL: Standard tessellation language
cw: Clockwise
ccw: Counterclockwise
IsL/NA: Upper incisor proclination angle
IiL/NB: Lower incisor proclination angle
ICC: Intraclass correlation coefficient
n: Number
yrs: Years
SD: Standard deviation
